# Supplementary material for: Does the Recovery of Respiratory Viruses Impact Pulmonary Function at Baseline and 1-, 6-, and 12-Month Follow-Up in People Living with HIV and Pneumonia?
Source: Viruses. 2024 Feb 23;16(3):344. doi: 10.3390/v16030344 (PMC10974101; doi:10.3390/v16030344)

Table S1. Frequency of causes of exclusion to the study in the three groups.

**Total, screened individuals**	**248**
**Total, excluded individuals**	183
**Reason for exclusion no. †**	
**High risk of loss of follow-up**	59
**Use of antibiotics for more than 72 hours**	57
**Living outside the metropolitan area**	51
**No fixed housing**	23
**Recent hospitalization**	26
**Contraindication for induced sputum or bronchoalveolar**	15
**Intubation on admission**	9
**No address**	9
**Steroid use**	8
**Active neoplasia or in treatment**	6
**Use of cytostatics**	5
**Significant chronic pneumopathy**	5
**Obstructive pneumonia**	4
**Neutropenia**	2

* Includes the 3 groups (HIV with pneumonia, HIV without pneumonia and pneumonia without HIV).
† Several reasons for exclusion overlap in some individuals.

Table S2. Severity of airflow limitation (FEV1/FVC < LLN) at admission.

% of FEV1 of predicted	HIV/pneumonia	HIV	pneumonia	Total
FEV1 ≥ 80%	1	0	0	1
FEV1 50% to 79%	3	4	0	7
FEV1 30% to 49%	1	1	3	5
FEV1 < 30%	2	0	0	2

Table S3. Microbiological identification in individuals with community-acquired pneumonia.

	**HIV/pneumonia n= 27**	**HIV/pneumonia n= 7**	**Total, n= 34**
**Individuals with at least one respiratory virus, n (%) ***	14/27 (51.8)	4/7 (57.1)	18/34 (52.9)
**HRV + *M. tuberculosis* †**	2		
**HRV + *M. tuberculosis* + *H. influenzae***	1		
**HRV + *P. jirovecii***	2		
**HRV + *P. jirovecii* + *S. pneumoniae***	1		
**HRV + *S. aureus***	2		
**HRV + BGN ‡**	1		
**HRV + FluA + polymicrobial §**		1	
**HRV only**	1	2	
**PIV1 + *M. tuberculosis***	1		
**PIV1 + *S. pneumoniae***	1		
**PIV3 only**		1	
**FluA + *M. tuberculosis***	1		
**FluA + polymicrobial**			
**MPV + *S. aureus***	1		
**Coronavirus NL63 + polymicrobial**	1		
**Adv + *S. pneumoniae***	1		
Mycobacterium tuberculosis* n *(%)	11/27 (40.7)	0	11/34 (32.4)
***M. tuberculosis* alone**	4 (14.8)		
***M. tuberculosis* + BGN**	2 (7.4)		
Pneumocystis jirovecii* n *(%)	6/27 (22.2)	0	6 (17.6)
***P. jirovecii* alone**	3 (11.1)		
**Gram-negative bacilli n (%)**	4/27 (14.8)	0	4 (11.7)
**BGN only**	3 (11.1)		
Streptococcus pneumoniae* n *(%)	3/27 (11.1)	0	3 (8.8)
***S. pneumoniae* alone**	1 (3.70)		
Sthaphylococcus aureus* (MSSA*)* n *(%)	2/27 (7.4)	0	2 (5.9)
***S. aureus* alone**	1 (3.70)		
**Polymicrobial n (%)**	2/27 (7.4)	1 (14.2)	3 (8.8)
**Polymicrobial alone**	1 (3.7)		
Haemophilus influenza,* n *(%)	1/27 (3.7)	0	1 (2.9)
**Germ-free, n (%)**	1/27 (3.7)	3 (42.9)	4 (11.7)

* The sum total of the percentages is greater than 100 due to the presence of several cases with multiple microorganisms.

† HRV: rhinovirus, PIV: parainfluenza virus, Flu: influenza virus, NL63: coronavirus, MPV: metapneumovirus, RSV: respiratory syncytial virus, AdV: adenovirus.

‡ BGN: Gram-negative bacilli.
§ Polymicrobial refers to the identification of 3 or more different types of bacteria in the same sample.

Table S4. Microbiological isolates by the presence of respiratory viruses.

Microorganism	No respiratory virus	Yes respiratory viruses	Total
Mycobacterium tuberculosis	6	5	11
Pneumocystis jirovecii	3	3	6
Gram negative bacilli	3	1	4
Streptococcus pneumoniae	2	1	3
Methicillin susceptible *Staphylococcus aureus*	1	1	2
Polymicrobial	0	3	3
Haemophilus influenzae	0	1	1

Table S5. Clinical diagnosis of individuals in the HIV+/pneumonia- group.

**Clinical diagnosis**	Total n= 31
Syphilis	5
Ambulatory control without comorbidity	5
Acute diarrhea	3
Headache of non-infectious origin	2
Upper respiratory infection	2
Adenopathies	2
Nodal tuberculosis	2
Heart failure	2
Intestinal tuberculosis	1
Cerebral Toxoplasmosis	1
Mycobacterium avium ganglionarum	1
Paracoccidioides brasiliensis	1
Disseminated histoplasmosis	1
Acute hepatitis C	1
Hepatic toxicity due to drugs	1
Meningitis	1
Urolithiasis	1
Urinary tract infection	1
Pelvic inflammatory disease	1
Herpes zoster	1
Esophageal Candidiasis	1
Cytomegalovirus colitis	1
Cryptosporidiosis	1
Urticaria	1

* Several individuals presented simultaneously more than one pathology.

Figure S1. Spirometric variables (FEV1 and FVC) according to the percentage of predicted at admission and at 1-, 6- and 12-month follow-ups.

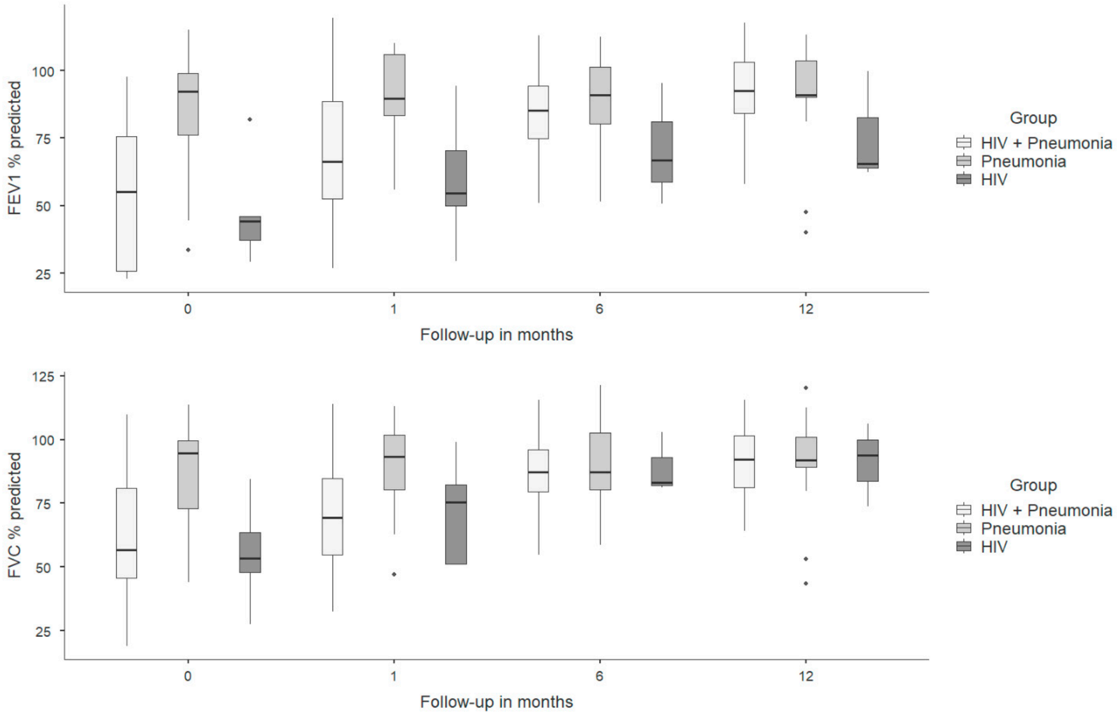

Supplement: Supplementary file 1 [file viruses-16-00344-s001.zip › viruses-2810915-supplementary.pdf]
